# LOCALIZATION OF Β-AMYLOID PLAQUES IN THE BRAINS OF WAR VETERANS, PARTICIPANTS OF THE DOD-ADNI STUDY

**DOI:** 10.1093/geroni/igad104.2236

**Published:** 2023-12-21

**Authors:** Stanislav Kolpakov Nikitin, Arseniy Yashkin, Igor Akushevich

**Affiliations:** Duke University, Durham, North Carolina, United States; Duke University, Durham, North Carolina, United States; Duke University, Durham, North Carolina, United States

## Abstract

We examined the allocation of β-amyloid (Aβ) plaques in the brains of Veterans, long-time survivors of traumatic brain injury (TBI), and those, who did not report brain trauma, and compared it with a spatial distribution of Aβ plaques in the brains of participants of the ADNI program as well as with the pattern of the spatial distribution of Aβ in the brains of participants of ADNI with Alzheimer’s disease. This study included 675 community-dwelling male participants from the ADNI and DoD-ADNI databases (137 veterans, 131 cases of TBI, and 123 AD cases) 62 years old or older. We performed regression analysis, using a pseudo-randomization algorithm, and propensity-score inverse-probability weighting to equalize the subsamples for fourteen outcomes, 12 standardized uptake value ratio variables, and 0.79 and 1.11 cutoffs. Race, educational level, geriatric depression score, age when florbetapir-18 (18F) PET scans were performed, APOE genotype, and Modified Hachinski Ischemic Score were used as predictors. The pattern common for AD showed the highest levels of β-amyloid in the cingulate cortex as well as in the frontal, parietal, and temporal lobes. Veterans have shown a statistically significant increase of 18F concentration in the cerebellum gray matter along with a lower concentration of it in the whole cerebrum as well as in neocortical regions than patients, who did not participate in combats. The continuous exposition to micro-TBI events, which does not necessarily suppose the loss of consciousness or severe contusion likely can explain the high concentration of 18F in cerebellum gray matter in ex-combatants.

